# Supplementary material for: Characterizing the role of RSD-6 in the biogenesis of virus-derived small interfering RNAs and the modulation of viral pathogenesis
Source: J Virol. 2025 Dec 12;100(1):e01516-25. doi: 10.1128/jvi.01516-25 (PMC12817956; doi:10.1128/jvi.01516-25)
Supplement: Supplemental figures — Figures S1 to S4. [file jvi.01516-25-s0001.pdf]

Figure S1. Distinct abundance and composition of siRNAs derived from Orsay virus RNA1 and RNA2.

Shown are the size distribution and abundance of primary and secondary vsiRNAs mapped to Orsay virus RNA1 (ovRNA1) and RNA2 (ovRNA2) genomic segments. Read counts for siRNAs are normalized to one million total miRNA reads.

Figure S2. siRNAs mapped to OrV RNA1 exhibit the same pattern of distribution among N2 and mutant animals as indicated. Shown here is the mapping of 22-24 nt sense (red) and antisense (blue) vsiRNAs to the full-length OrV RNA1. The sequencing libraries were constructed using 5' P-dependent protocol. The relative abundance was normalized to one million total miRNAs.

Figure S3. *C. elegans* RSD-6 and *C. briggsae* RSD-6 exhibit distinct function domain arrangements. A. AlphaFold3-predicted structures of wildtype *C. elegans* RSD-6 viewed in UCSF Chimera. C. AlphaFold3-predicted structures of wildtype *C. briggsae* RSD-6. C. Wildtype *C. elegans* RSD-6 superimposed on wildtype *C. briggsae* RSD-6 with the Tudor domain aligned in UCSF Chimera.

Figure S4. Schematic presentation of antiviral RNAi pathway in *C. elegans*.

Upon RNA virus infection, viral genome replication produces dsRNA intermediates which are detected by DRH-1 (Dicer-related RNA helicase 1). DRH-1 recruits, through interaction with dsRNA-binding protein RDE-4 (RNAi defective 4), DCR-1 (worm Dicer) to process viral dsRNAs into primary vsiRNAs. These vsiRNAs are then loaded onto RDE-1, which guides sequence-specific recognition of viral RNA. RDE-8 (RNAi defective 8), an endoribonuclease, cleaves target viral RNA. RDE-3, a nucleotidyltransferase, adds poly(UG) tails to the 3' ends of cleaved viral RNAs, yielding template for the production of secondary vsiRNAs by RRF-1. These secondary siRNAs are then loaded onto WAGO (worm-specific Argonaute) proteins, which mediate robust antiviral silencing. RSD-6 acts downstream of secondary vsiRNA biogenesis, contributing to secondary vsiRNA-mediated antiviral defense.

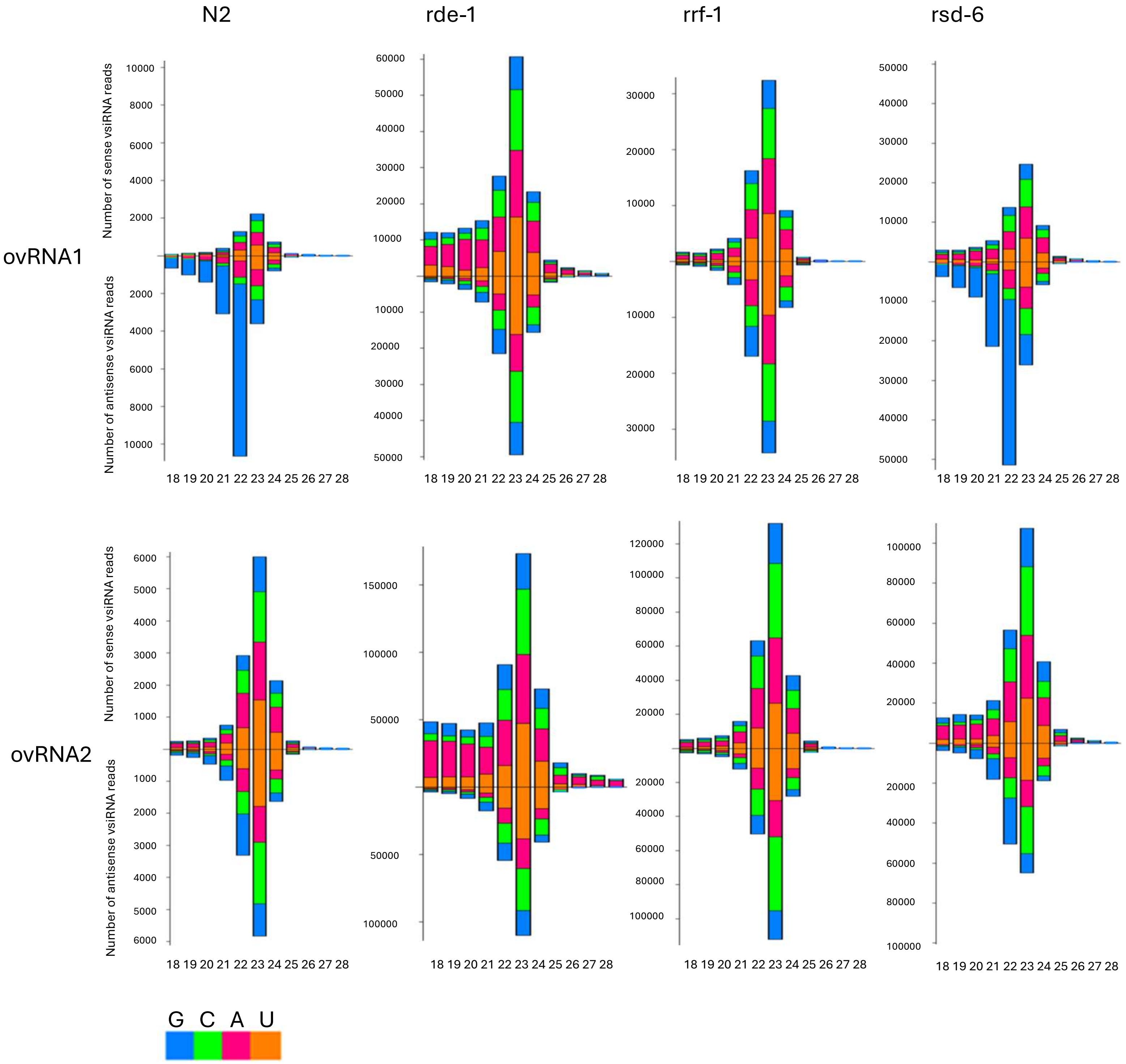
N2
rde-1
rrf-1
rsd-6
ovRNA1
ovRNA2
Number of sense vsiRNA reads
Number of antisense vsiRNA reads
18 19 20 21 22 23 24 25 26 27 28
G C A U

Figure S1

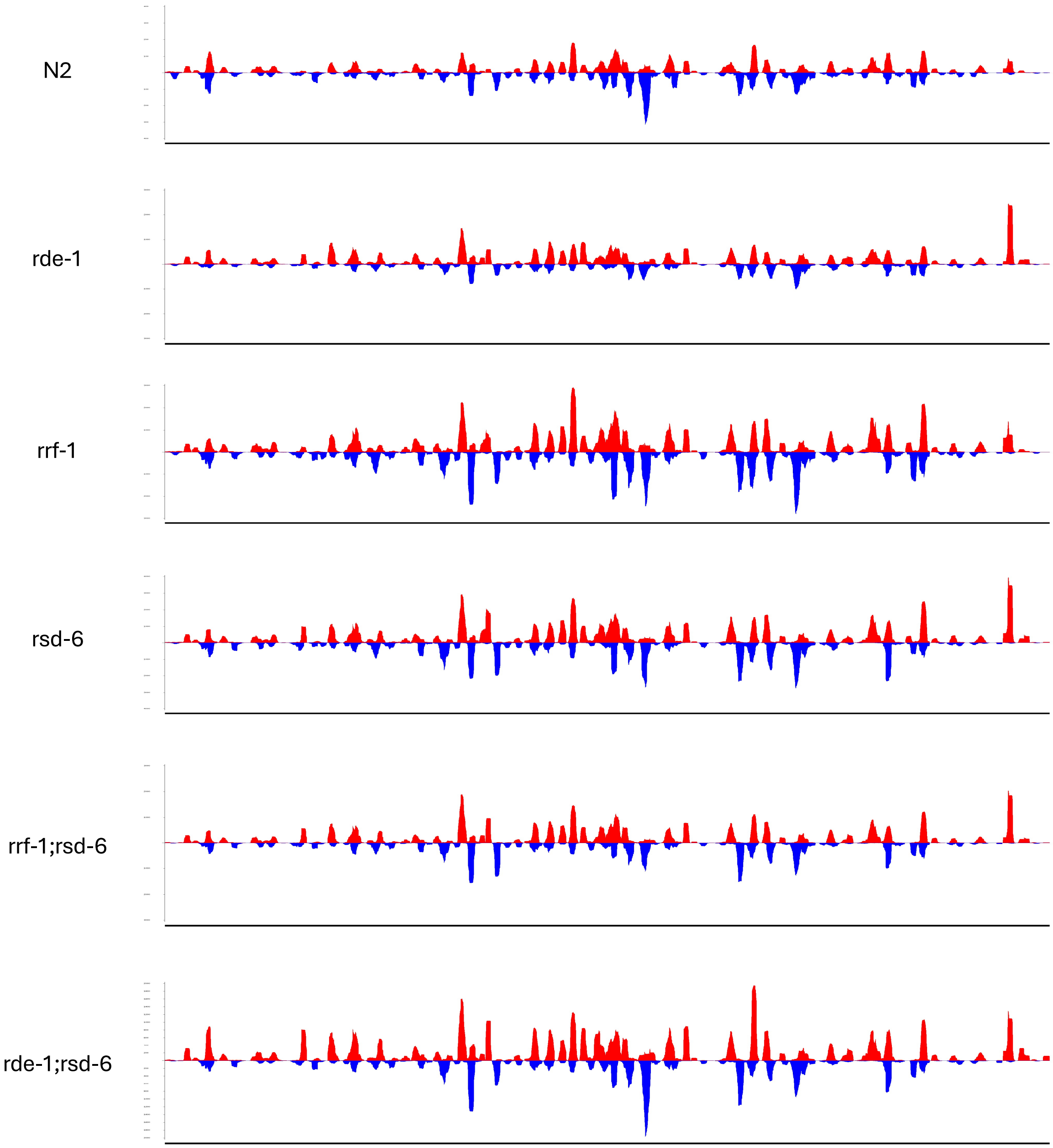

Figure S2.

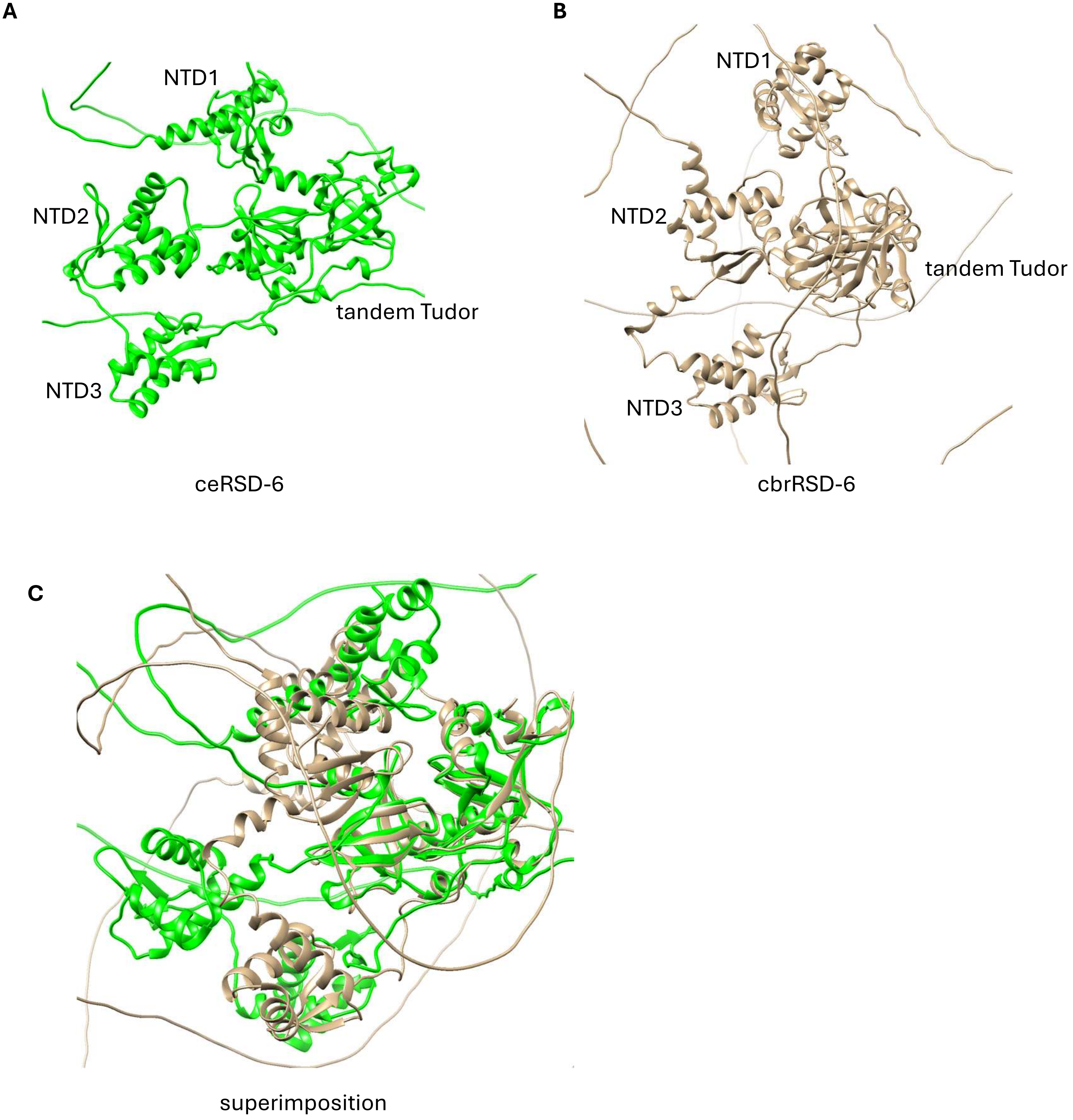
A
NTD1
NTD2
tandem Tudor
NTD3
ceRSD-6
B
NTD1
NTD2
tandem Tudor
NTD3
cbrRSD-6
C
superimposition

Figure S3.

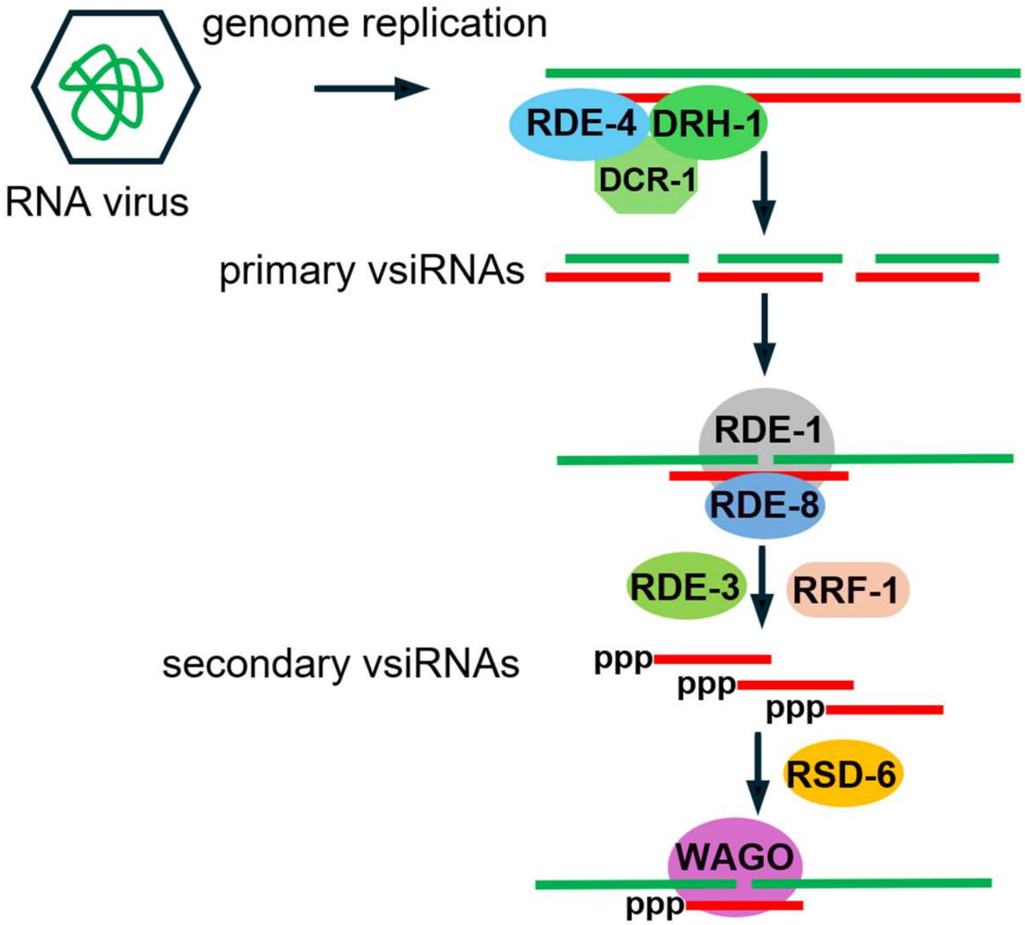
genome replication
RNA virus
RDE-4
DRH-1
DCR-1
primary vsiRNAs
RDE-1
RDE-8
RDE-3
RRF-1
secondary vsiRNAs
ppp
ppp
ppp
RSD-6
WAGO
ppp

Figure S4.
