# Supplementary material for: Characterizing the role of RSD-6 in the biogenesis of virus-derived small interfering RNAs and the modulation of viral pathogenesis
Source: J Virol. 2025 Dec 12;100(1):e01516-25. doi: 10.1128/jvi.01516-25 (PMC12817956; doi:10.1128/jvi.01516-25)
Supplement: Supplemental tables — Tables S1 to S3. [file jvi.01516-25-s0002.docx]

Table S1. Number of reads obtained for each sequencing library constructed.

| **siRNA sample** | **number of reads** |
| --- | --- |
| N2 | 39,349,281 |
| N2. phosphatase treated | 16,949,941 |
| rde-1 | 22,058,757 |
| rde-1. phosphatase treated | 25,825,223 |
| rrf-1 | 32,672,241 |
| rrf-1. phosphatase treated | 41,812,983 |
| rsd-6 | 28,298,955 |
| rsd-6. phosphatase treated | 36,659,974 |
| rde-1;rsd-6 | 8,967,274 |
| rde-1;rsd-6. phosphatase treated | 21,929,469 |
| rrf-1;rsd-6 | 9,714,639 |
| rrf-1;rsd-6. phosphatase treated | 13,981,723 |

Table S2. Nematode strains used in this study

| Laboratory  name | Strain and allele name | genotype | Source |
| --- | --- | --- | --- |
| N2 | N2 |  | CGC |
| RB786 | *rde-1(ok569)* | [rde-1(ok569)V] | CGC |
| RB798 | *rrf-1 (ok589)* | [rrf-1 (ok589) I ] | CGC |
| RL4350 | *rde-4 (yt4350)* | [rde-4 (yt4350) III ] | Lab  strain |
| RL622 | *rsd-6 (kd622)* | [rsd-6 (kd622) I ] | Lab  strain |
| WM286 | *rde-3 (ne3370)* | [rde-3 (ne3370) I ] | CGC |
| RB2519 | *drh-1 (ok3495)* | [drh-1 (ok3495) IV] | CGC |

Table S3. DNA and RNA Oligos used in this study.

| Oligo Name | Sequence | Notes |
| --- | --- | --- |
| rsd6gRNA1 | AGAGACCCUUCUCCACGACG | crRNA for generating rsd-6 knockout |
| rsd6gRNA2 | AGGUCUCAUGGCUAAGGCCA | crRNA for generating rsd-6 knockout |
| rsd-6 ssODN | ACATGAATGAAAAAGAGCTGGCGGATTCTGTTTTTAGAGTTCTCATAGCTAATGACGCTGAAACAATTATGGAGACATAAGAGGACGTCCAGAC | Single-stranded oligo DNA donor for generating rsd-6 knockout |
| rsd-6-uns-11-F | actcgacATGAATGAAAAAGAGCTG | For allele kd622 genotyping |
| rsd-6 1430m | AGTCTTACTCCCCGACGCAT | For allele kd622 genotyping |
| rde4gRNA1 | UCCCCUCAUGGUACUAGAAG | crRNA for generating rde-4 knockout |
| rde4gRNA2 | ACGCUCGGUUCUCUCAUUCU | crRNA for generating rde-4 knockout |
| Rde-4 ssODN | GATGTTTCTGAAGAAAACTCCCCTCATGGTACTAGATGAGAGAACCGAGCGTTTCTTGGTTATATGCACG | Single-stranded oligo DNA donor for generating rsd-6 knockout |
| 4350-1F | GTTTCTTTAAACGAGATCCGC | For allele yt4350 genotyping |
| rde4i-R | TAGCCTCTTCCGCCGATGAAC | For allele yd4350 genotyping |
| rde-1.36F | CCGGTATGATCAATTATTAGCAGC | For allele ok569 genotyping |
| rde-1.36R | ACTAATTTACAAGCACTTACATTGG | For allele ok569 genotyping |
| rde-3Bbs | AAAGATCAGCGAAGTCCAG | For allele ne3370 genotyping |
| rde-3ear | TGAACAATCTTGCCAGTGG | For allele ne3370 genotyping |
| 589XcmI | GTTGTCTCGTCTGAGGTCTCG | For allele ok589 genotyping |
| rrf-1 BamHI | TCCAAATCGGATCCAGCCAT | For allele ok589 genotyping |
| FR1gfp.F | AGGTCTTTGTTTCCTGTCTCGTG | qPCR primer for FR1gfp RNA1 detection |
| FR1gfp.R | GCCTTCGACACGGTCGCAA | qPCR primer for FR1gfp RNA1 detection |
| FR1gfp.subF | GCGGAATTCACCGGGGTGGTGCCCATCCT | qPCR primer for FR1gfp RNA3 detection |
| FR1gfp.subR | TGCAGATGAACTTCAGGGTCAGC | qPCR primer for FR1gfp RNA3 detection |
| OrV.FF | CGCACGGATACTGGCTTACC | qPCR primer for OrVRNA1 detection |
| OrV.RR | AACACCAAGACGCTTCCAAGATT | qPCR primer for OrVRNA1 detection |
| act-1.F | TAGACAATGGATCCGGAATGTGC | act-1 primer in qPCR |
| act-1.R | GACGTACGAGTCCTTCTGTCC | act-1 primer in qPCR |
